# Naturally-occurring myopia and loss of cone function in a sheep model of achromatopsia

**DOI:** 10.1038/s41598-020-76205-z

**Published:** 2020-11-09

**Authors:** Maya Ross, Ron Ofri, Itzhak Aizenberg, Mazen Abu–Siam, Oren Pe’er, Dikla Arad, Alexander Rosov, Elisha Gootwine, Hay Dvir, Hen Honig, Alexey Obolensky, Edward Averbukh, Eyal Banin, Liat Gantz

**Affiliations:** 1 Koret School of Veterinary Medicine, Hebrew University of Jerusalem, Rehovot, Israel; 2 Abu-Siam Veterinary Clinic, Rahat, Israel; 3 Institute of Animal Science, Agricultural Research Organization, Volcani Center, Rishon LeZion, Israel; 4 Department of Ophthalmology, Hadassah-Hebrew University Medical Center, Jerusalem, Israel; 5 Department of Optometry and Vision Science, Hadassah Academic College, 37 Haneviim St., Jerusalem, 9101001 Israel

**Keywords:** Experimental models of disease, Eye diseases

## Abstract

Achromatopsia is an inherited retinal disease characterized by loss of cone photoreceptor function. Day blind *CNGA3* mutant Improved Awassi sheep provide a large animal model for achromatopsia. This study measured refractive error and axial length parameters of the eye in this model and evaluated chromatic pupillary light reflex (cPLR) testing as a potential screening test for loss of cone function. Twenty-one *CNGA3* mutant, Improved Awassi, 12 control Afec-Assaf and 12 control breed-matched wild-type (WT) Awassi sheep were examined using streak retinoscopy and B-mode ocular ultrasonography. Four *CNGA3* mutant and four Afec-Assaf control sheep underwent cPLR testing. Statistical tests showed that day-blind sheep are significantly more myopic than both Afec-Assaf and WT Awassi controls. Day-blind sheep had significantly longer vitreous axial length compared to WT Awassi (1.43 ± 0.13 and 1.23 ± 0.06 cm, respectively, p < 0.0002) and no response to bright red light compared to both controls. Lack of response to bright red light is consistent with cone dysfunction, demonstrating that cPLR can be used to diagnose day blindness in sheep. Day-blind sheep were found to exhibit myopia and increased vitreous chamber depth, providing a naturally occurring large animal model of myopia.

## Introduction

Congenital achromatopsia (ACHM) is an inherited retinal disease, characterized by loss of cone photoreceptor function. Most patients are legally blind from birth, suffering from severe impairment of visual acuity and loss of color vision, as well as nystagmus and severe photophobia^1,2^. In most cases, ACHM is caused by mutations in genes coding for a cone cyclic nucleotide-gated (CNG) ion channel, specifically in its α (CNGA3)^3,4^ or β (CNGB3)^5,6^ subunits. These lead to channel closure failure, inability of the photoreceptor to hyperpolarize, and cone dysfunction.

One of the most famous cases of ACHM has been documented in the Pingelap and Mokil Atolls in the Pacific Ocean, and later described by Oliver Saks in his popular book, The Island of the Colorblind^7^. These islands were devastated by a typhoon in 1775. There were only 20 survivors, one of them the future king, who was a carrier of a *CNGB3* mutation. Since then, cases of *CNGB3* ACHM began appearing amongst the islanders, with twentieth-century epidemiological studies showing a disease prevalence of about 10%^8–11^, and another 30% of the population being carriers. Interestingly, a majority of ACHM cases were also affected by high myopia (− 5 to − 17 Diopters, (D)), with 50–85% of patients affected by both disorders^12,13^. Subsequent studies demonstrated a high prevalence of myopia in *PDE6C* ACHM (myopia > 6D in 63% of patients)^14^, and increased susceptibility to form-deprivation myopia in a mouse model of *GNAT2* ACHM^15^. Increased prevalence of high myopia has also been reported in other cone dysfunction retinopathies^16–19^. Contrary to these reports, other studies report a high prevalence of hyperopia^20^ or a combination of both^5,21,22^.

In recent years, there is increased recognition that another clinical manifestation of cone dysfunction is loss of pupillary light reflex (PLR) in response to high intensity red light. The chromatic pupillary light reflex (cPLR) test is based on studies showing that PLR to dim blue light, high intensity red light and high intensity blue light originates in the rod pathway, cone pathway and intrinsically photosensitive retinal ganglion cells (ipRGCs), respectively^23,24^. This has led to development of protocols to assess rod, cone and ipRGC contribution to the PLR in humans and animal models, using red and blue light stimulation of varying intensities delivered by Ganzfeld^25^, multifocal Ganzfeld^26^, or diode-based light^27^. Using this methodology, it has been shown that in Leber’s Congenital Amaurosis patients there is selective loss of PLR in response to dim blue light, but not in response to bright red or blue light, demonstrating selective loss of rod function^28^. Similar results were obtained in retinitis pigmentosa patients^26,29^. Conversely, loss of PLR in response to bright red light, but not to dim or bright blue light, has been demonstrated in achromatopsia patients^24,30^.

In 2010 we reported on congenital day blindness in the Improved Awassi breed of sheep^31^. This autosomal recessive hereditary disease was determined to be caused by a C → T substitution leading to a premature stop codon at residue 236 of the ovine *CNGA3* gene (c.706C > T, p.R236*), thus making affected sheep a naturally-occurring large animal model for human *CNGA3* ACHM ^32^. Later, a second (missense) mutation resulting in day blindness in sheep was identified in the same gene (c.1618G > A, p.Gly540Ser)^33^. Like most non-primate mammals, the retina of a sheep contains a specialized region called an “area centralis”, rather than a fovea. Like the fovea, this area contains the animals’ highest concentration of cone photoreceptors; however, unlike the fovea, rods outnumber cones even in the area centralis. In sheep, for example, the retina contains approximately 35 times more rod photoreceptors than cone photoreceptors^34^, and nine times more rod photoreceptors than cone photoreceptors in the area centralis^35^. Mutations in *CNGA3* affect all of the cone photoreceptors throughout the retina of day-blind sheep.

Subsequently, we used these *CNGA3* mutation models for successful gene therapy trials, based on subretinal injections of an adeno-associated virus carrying the *CNGA3* transgene under the control of a cone-specific promoter^33,36,37^. Treatment restored cone function and photopic (daytime) vision within a few days in all treated sheep, as demonstrated both behaviorally and electroretinographically (ERG), with the oldest treated sheep still visual after more than 9 years^38^. Based on our results, as well as work of other teams using murine and canine models of the disease^39–43^, Phase I/IIa clinical trials have been approved in human ACHM patients (NCT02935517 and NCT02610582), demonstrating the translational relevance of our work with this large animal model of *CNGA3* ACHM .

Though very successful, studies using large animal models of ACHM, such as sheep and dogs, are very laborious. Animals have to be anesthetized for ERG recordings^44,45^, and maze testing is time consuming and requires a special setup^33,46^. Therefore, our first aim was to develop rapid, non-invasive, and easily conducted tests for screening research animals for cone dysfunction. Our second aim was to further characterize the ovine *CNGA3* ACHM model, as we are continuing work on developing intravitreal delivery of the viral vector^47^. To this end, we conducted cPLR and refraction (supplemented by ultrasonography) testing of wild type (WT) control sheep of two breeds, as well as *CNGA3* day blind sheep.

## Results

### Refraction

**Figure 1:**
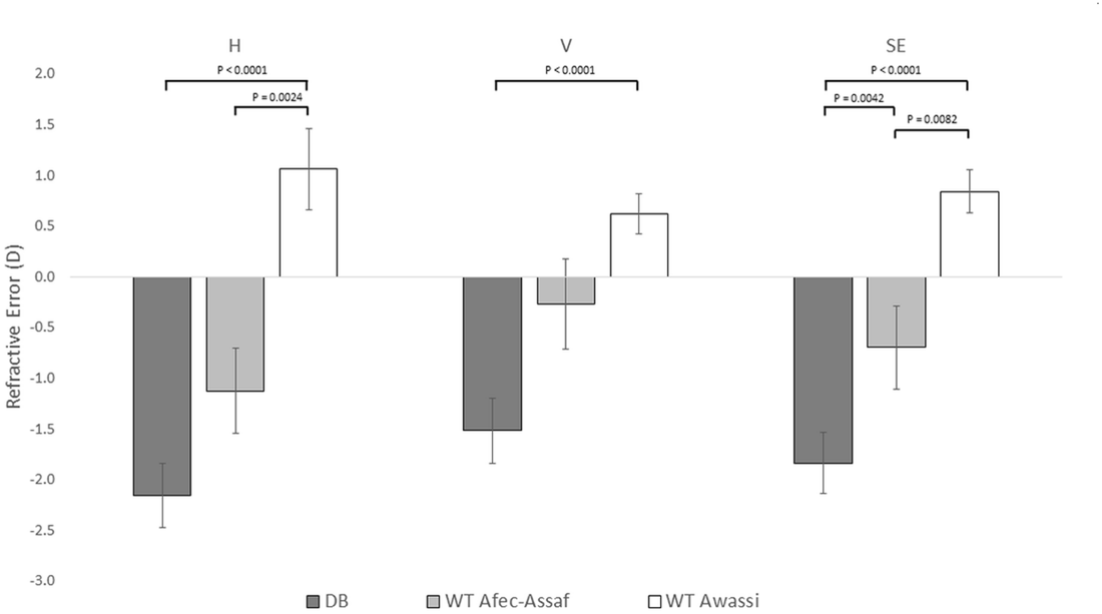
Refractive errors of day-blind sheep compared to two cohorts of wild type sheep. The mean and standard error of the mean refractive error as determined using a streak retinoscope for the horizontal (H) and vertical (V) meridians and the spherical equivalent refractive error (SE) in the left eyes of Improved Awassi day-blind sheep (cohort 1, dark gray), Afec-Assaf wild-type sheep raised under the same husbandry conditions (cohort 2, light gray), and the breed-matched Awassi wild-type sheep (cohort 3, white). Pairwise comparisons were made using the Mann–Whitney nonparametric test and p-values are specified in comparisons that yielded a significant result (p < 0.017).

Figure 1 presents the mean and standard error of the refractive error in each sheep cohort for the horizontal (H) and vertical (V) meridians, as well as the calculated spherical equivalent (SE) refractive error. The right and left eye refractive errors for the horizontal and vertical meridians were significantly correlated (H, Spearman's ρ = 0.78, p < 0.0001; V, Spearman's ρ = 0.78, p < 0.0001). In addition, no significant differences were found between the refractive errors of the left eyes in the treated and untreated day- blind sheep of cohort 1 (P > 0.05). Therefore, in accordance with statistical guidelines recommending that data from both eyes should not be combined^48,49^, and because the right eyes of some sheep in Improved Awassi day-blind sheep (cohort 1) were used in a gene therapy study^36,38^, the refractive error of only the left eyes was used in subsequent analyses.

The mean refractive error in the horizontal and vertical meridians of the Improved Awassi day-blind and Afec-Assaf WT sheep (cohorts 1 & 2, respectively) were myopic (defined as a refractive error greater than − 0.50 D in the least myopic eye^50^), while the WT Local Awassi sheep (cohort 3) were slightly hyperopic (defined as a refractive error greater than + 0.50 D in the least hyperopic eye^51^) in both meridians (H meridian: − 2.15 ± 0.31 D, − 1.12 ± 0.42 D and 1.06 ± 0.42 D; V meridian: − 1.52 ± 0.29 D, − 0.27 ± 0.38 D and 0.62 ± 0.38 D in cohorts 1, 2 and 3 respectively). There was a significant effect of cohort type on all three refraction parameters (V meridian, p < 0.0003; H meridian, and SE refractive error p < 0.0001). Post hoc testing indicated that the Improved Awassi day-blind sheep (cohort 1) were significantly more myopic than the Afec-Assaf WT sheep raised under the same husbandry conditions (cohort 2) in the SE refractive error (p < 0.005). Furthermore, the Improved Awassi day-blind sheep (cohort 1) also differed significantly from the breed matched Local Awassi WT sheep (cohort 3) in all refraction parameters, as the former were myopic and the latter were hyperopic (all: p < 0.0001). Finally, Afec-Assaf WT sheep (cohort 2) differed from the Local Awassi WT sheep (cohort 3), as the former were myopic and the latter were hyperopic, with significant differences between the two cohorts in the horizontal meridian and in the SE refractive error (p < 0.003 and p < 0.01, respectively).

### Ocular ultrasonography

**Figure 2:**
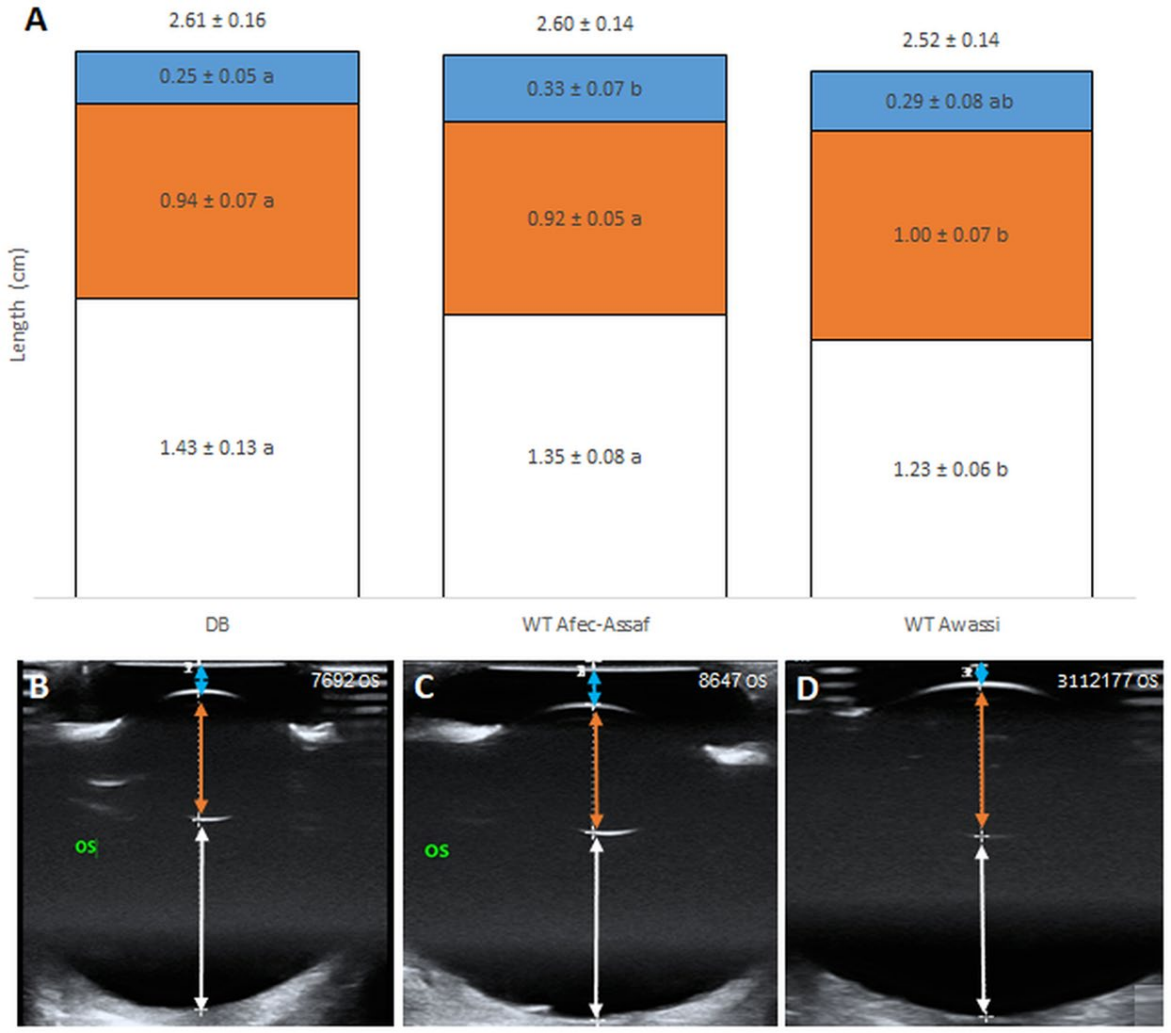
Globe axial length, anterior chamber depth, lens axial length and vitreous chamber depth of the three study cohorts. **(A)** Mean length (± standard error) of each parameter in the left eyes of the three cohorts. White bars represent the vitreous chamber depth, orange bars represent the lens axial length, and blue bars represent the anterior chamber depth. The mean globe axial length is presented above the stacked bars. For each parameter, bars marked with different letters are significantly different (p < 0.017). **(B–D)** One representative scan from each cohort. The vitreous chamber is marked by a white arrow, the lens is marked by an orange arrow and the anterior chamber is marked by a blue arrow. **(B)** Cohort 1—Improved Awassi day-blind sheep, **(C)** Cohort 2—Husbandry-matched control group of Afec-Assaf WT sheep and, **(D)** Cohort 3—Breed-matched control group of Awassi WT sheep. *WT* wild type, *DB* day blind.

Ocular ultrasonography was used to measure four parameters: globe axial length, anterior chamber depth, lens axial length and vitreous chamber depth in the three cohorts. Most ultrasonographic measurements of left and right eyes of each sheep were significantly correlated (globe axial length, Spearman's ρ = 0.79, p < 0.0001; lens axial length, Spearman's ρ = 0.58, p < 0.0001; vitreous chamber depth, Spearman's ρ = 0.83, p < 0.0001), therefore only the left eyes were used in subsequent analyses. Figure 2 presents the mean length (cm) of the four parameters of the left eyes in all cohorts, and a representative scan of one eye from each cohort. Globe axial length did not differ significantly between the three cohorts (Fig. 2A, top row). However, the inner compartments' length was significantly different among the three cohorts. Overall, the most notable finding was a significant elongation of the vitreous chamber in the day-blind cohort compared to the breed-matched WT Awassi cohort (Fig. 2A, white columns; p < 0.0002). There was a significant effect of cohort type on vitreous chamber depth (p < 0.0001), lens axial length (p < 0.02) and anterior chamber depth (p < 0.004). Post hoc testing demonstrated that the Improved Awassi day-blind sheep (cohort 1) differed significantly from the breed-matched Local WT Awassi sheep (cohort 3) in the vitreous chamber depth (p < 0.0002) and in the lens axial length (Fig. 2A, white and orange columns; p = 0.017). Improved Awassi day-blind sheep (cohort 1) also differed significantly from the WT Afec-Assaf sheep raised under the same husbandry conditions (cohort 2) in anterior chamber depth (Fig. 2A, blue columns; p < 0.02). Finally, the two WT control cohorts (cohorts 2 & 3) differed significantly from each other in vitreous chamber depth (p < 0.02) and lens axial length (Fig. 2A, white and orange columns; p < 0.02).

### Chromatic pupil light reflex (cPLR)

**Figure 3:**
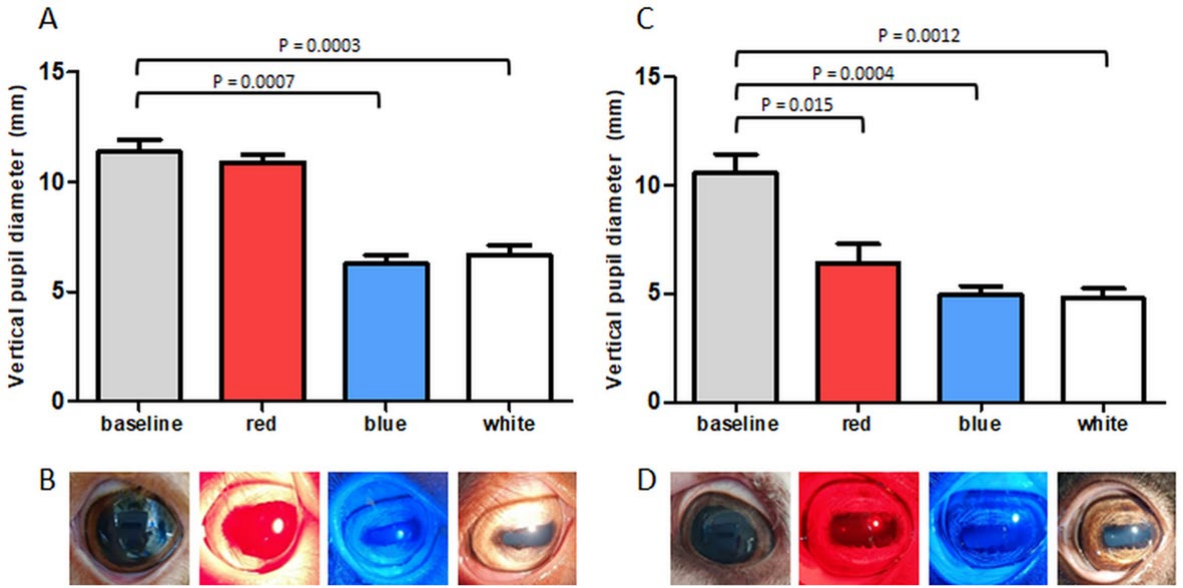
Pupil constriction in response to bright red, blue and white light in day-blind and wild type sheep. **(A)** Mean vertical pupil diameter (+ standard error) of Improved Awassi day-blind sheep (cohort 1, n = 4), at baseline and after bright red (630 nm), blue (480 nm) and white illumination. **(B)** Representative images from a single Improved Awassi day-blind sheep, showing mydriasis in dim light (baseline conditions). The pupil does not constrict in response to bright red light, but does constrict in response to bright blue and white light. **(C)** Mean vertical pupil diameter (+ standard error) of Afec-Assaf WT, husbandry-matched control sheep (cohort 2, n = 4), at baseline and after red, blue and white illumination. **(D)** Representative images from an exam of a single Afec-Assaf WT control sheep. Unlike the animal in panel **(B)**, the pupil constricts in response to red light. P-values are specified for significantly different results.

Baseline vertical pupil diameter was measured in both eyes of four Improved Awassi day-blind and four WT Afec-Assaf control sheep (cohorts 1 & 2, respectively) under dim light conditions. Subsequently, the vertical pupil diameter in response to bright red, blue and white light was measured. Mean vertical pupil diameters and a representative image of one eye from each cohort are presented in Fig. 3. Since the measurements in the left and right eyes were significantly correlated (Spearman's ρ = 0.98, p < 0.0001), only the left eyes were used for statistical analyses.

Compared to baseline values, the pupils of Improved Awassi day-blind animals (cohort 1) constricted significantly in response to bright blue and white, but not bright red, light (vertical pupil diameter: baseline 11.36 ± 1.97 mm, red 10.72 ± 1.16 mm, blue 6.16 ± 1.27 mm and white 6.85 ± 1.40 mm, Fig. 3A,B), demonstrating loss of cone function in these animals. On the other hand, WT, husbandry-matched control pupils (cohort 2) constricted significantly, compared to baseline values, in response to bright red, blue and white light (vertical pupil diameter: baseline 10.68 ± 0.93 mm, red 6.35 ± 1.00 mm, blue 4.91 ± 0.54 mm, and white 4.68 ± 0.42 mm, Fig. 3C,D). When comparing vertical pupil diameter, there was a significant difference between the two cohorts in response to red stimulation (p < 0.03), but no significant difference was observed at baseline (dim light) and in response to bright blue and white stimulation.

## Discussion

In the present study, we report on naturally occurring myopia in a sheep model of day-blindness. Our main finding is that Improved Awassi day-blind sheep are significantly more myopic compared to WT Afec-Assaf control sheep raised under the same conditions (which tend to be slightly myopic) and compared to Local Awassi WT breed-matched sheep (which are hyperopic) (Fig. 1). The myopia in the Improved Awassi day-blind sheep is likely due to the significant elongation of the vitreous chamber we report in this cohort (Fig. 2). We also report that there is a significant difference in the vertical pupil diameter in response to red stimulation of Improved Awassi day-blind sheep and WT Afec-Assaf control sheep (Fig. 3), providing evidence of absence of cone function in the former.

Myopia is the most common eye condition in the world^52^, reportedly affecting approximately 28% of the world population in 2016 with a projected increase in prevalence of up to 50% by 2050^53^. The prevalence of myopia is 90% in urban East Asia^54^ and roughly 40% in Western countries^55^. Myopia poses a substantial economic burden^56,57^, and is associated with other blinding comorbidities such as retinal detachment, subretinal neovascularization, dense cataract, and glaucoma that increase risks of severe and irreversible loss of vision^58^. Therefore, there is a great impetus and urgent need to find and develop animal models for investigating this important disease. To date, the only naturally occurring large animal model of the disease is the dog^59–61^. In the current study, we found that Improved Awassi day-blind sheep are significantly more myopic than WT sheep with significantly longer vitreous chamber depth. Due to the vitreous elongation, incoming light is focused in front of the retina, resulting in near sightedness, or myopia. A similar correlation between myopia and vitreous elongation has been reported in dogs^61^ and in humans^62^ as well as in form deprivation studies in chicks^63^, tree shrews^64^, marmosets^65^, guinea pigs^66^ and other species. In all of these studies, which aimed to develop an animal model to study the mechanisms underlying the pathogenesis of myopia, form deprivation was generated by optical diffusers placed in front of the eye during development, leading to degradation of the visual image formed on the retina. This resulted in vitreous elongation and myopia^63–66^. In our naturally occurring animal model of day-blind sheep, we observed a similar association between myopia and vitreous elongation, though the pathogenesis may be different from that observed in the form-deprivation studies. One possible reason for the myopic development in our day-blind sheep could be cone dysfunction^45^. Due to the fact that cones are tasked with high resolution vision, loss of cone input results in rod-mediated low resolution vision, similar to that enabled with optical diffusers^67^. Therefore, it would seem that vitreous elongation and the consequent myopia may develop not only due to optical degradation of the image formed on the retina, but also due to loss of afferent cone input to higher visual centers. Indeed, it has been shown that myopia is associated with anatomical changes in the visual cortex, including reduced functional connectivity density in visual centers^68,69^, smaller gray matter volume^70^ and reduced intracranial volume^71^. Similar cortical reorganization has been reported in humans with congenital rod monochromacy (cone absence)^72^. Thus, it is possible that loss of visual input to the cortex, be it due to cone dysfunction or due to optical degradation of the visual image, plays a role in the pathogenesis of vitreous elongation and myopia. Another possible reason for myopia in our animals could be peripheral optical degradation, as myopia development can be mediated by peripheral hyperopic blur or peripheral form deprivation in animal models with ablated foveas^73^. Yet another possible mechanism could be a linkage between genetic variants associated with achromatopsia that may also be associated with myopia, such as demonstrated in *CNGB3* which causes achromatopsia in humans^74^.

It should be noted that even though our cohort of day-blind sheep exhibited a myopic mean refractive error, two sheep were hyperopic. Similarly, in human studies, the refractive error of those affected with achromatopsia is not always myopic. In one study, eleven adults with X-linked achromatopsia exhibited a myopic refractive error, and eight of the nine children with autosomal recessive achromatopsia did not exhibit myopia^21^. In another, 32 (46%) of 69 participants had refractive errors ranging between + 2.00 and − 2.00 Diopters, 17 (25%) of 69 had myopia higher than − 2.00 Diopters, and 20 of 64 (31%) had hyperopia larger than + 2.00 Diopters^5^. Similarly, a recent study reported high myopia (> 6 diopters) in five (62.5%), mild hyperopia in two (25%), and myopic astigmatism in one (12.5%) of eight patients with *PDE6C*-Associated Achromatopsia^14^. Achromatopsia may be caused by mutations of different genes^75^. Different mutations and genes may dissimilarly affect visual development and subsequent visual processing leading to variations in the distribution of refractive errors in this population.

In our study, two unaffected control groups were examined. Cohort 2 consisted of Afec-Assaf sheep raised under the same husbandry conditions as the day-blind Improved Awassi sheep. Cohort 3 consisted of breed-matched Local Awassi sheep that were raised in a different environment than the experimental, day-blind Awassi sheep. We found significant differences in both refractive errors and intraocular dimensions between the two cohorts (Figs. 1, 2). We are unable to determine whether the differences stem from breed or environmental differences. Breed differences in refractive errors have been reported in other species including dogs^76^, cats^77^, horses^78^. Similarly, environmental conditions have been reported to affect the refractive error in humans^79^ and cats^80^. Nonetheless, we believe the fact that the refractive errors and intraocular dimensions of our experimental, Improved Awassi day-blind sheep (cohort 1) differed significantly from those of both control cohorts makes our results more robust. In this context it should be mentioned that Piggins and Phillips (1996) refracted 36 sheep and found them to be slightly hyperopic (mean 1.2D), with only one myopic sheep in their cohort^81^. Interestingly, the same authors analyze the description of the sheep in Lewis Carrol's “Through the Looking Glass and What Alice Found There” and propose that the sheep in the book may have been myopic^82^.

cPLR testing is increasingly used to differentiate between outer and inner retinal diseases (based on lack of response to bright red and blue light, respectively) and to diagnose cone dysfunction based on lack of response to bright red light^24,26,29,30^. Our results demonstrate significant attenuation of PLR in response to bright red light in affected sheep (Fig. 3), indicating cone dysfunction. As cPLR is a non-invasive test that can be performed rapidly and easily in alert patients, we propose that it can serve as a screening test in small and large animal models of achromatopsia^43,83^, as well as non-verbal human patients such as babies^84^. It has yet to be determined whether cPLR could also be used to evaluate the efficacy of gene therapy in achromatopsia patients. It is noteworthy that cPLR testing has also been reported in ametropic patients, and it has been demonstrated that constriction to bright red light is most significant in hyperopes^85,86^.

There are limitations to this study, mainly with regards to the Local Awassi WT sheep (cohort 3). First, while sheep in cohorts 1 and 3 were of the Awassi breed, they were not bred in the same husbandry conditions and descended from different ancestry. Therefore, the differences in their refractive errors could also be attributed to the varying genetic and environmental conditions, though both cohorts were not involved in near visual tasks that can affect refractive development^79^. Second, due to the fact that cohort 3 sheep were located in a privately-owned farm, they were examined in unmasked conditions. These may have created an unintentional bias in the refractive and ultrasound measurements. Likewise, cPLR was evaluated in an unmasked fashion.

In conclusion, cone dysfunction in this animal model of achromatopsia may be confirmed by cPLR. The majority of day-blind sheep exhibited myopic refractive error and increased vitreous chamber depth, thus providing a naturally-occurring large animal model of the most common eye disorder .

## Methods

### Experimental animals

Three cohorts of sheep were examined in this study. Cohort 1 consisted of 21 *CNGA3* mutant, day-blind Improved Awassi sheep, of which three were male. Eight animals of cohort 1 previously underwent gene augmentation therapy in their right eye, while their left eye remained untreated as negative control^36,38^. Cohorts 2 and 3 consisted of WT control female sheep unaffected by the *CNGA3* mutation and included 12 Afec-Assaf breed and 12 Local Awassi sheep, respectively. Sheep in cohorts 1 and 2 were born and raised in open shed facility at the experimental flock of the Volcani Center, and their mean (± SD) ages were 32.9 ± 12.6 and 34.8 ± 20.4 months, respectively. Sheep in cohort 3 were born and raised outdoors by a private owner, and were all > 3 years old, though exact birth dates were unknown. Therefore, cohorts 2 and 3 served as husbandry-matched and breed-matched control groups, respectively. Experimental protocols were approved by the Volcani Center Animal Care and Use Committee (Approval no. IL 828/19) and conformed with the ARVO Statement on the Use of Animals in Ophthalmic and Vision Research.

### Refraction

The refractive error was determined during one experimental session for Improved Awassi day-blind and Afec-Assaf WT (cohorts 1 and 2, respectively), and a second session for WT Local Awassi sheep (cohort 3). The retinoscopist was masked regarding the genetic status of cohorts 1 and 2, and animals in this session were examined in random order to maintain masking. The retinoscopist was unmasked to the status of cohort 3, as all sheep in this cohort were WT.

Retinoscopy was performed by an experienced retinoscopist, a certified Israeli optometrist (LG) with over 15 years of experience. Prior to the experiment, the retinoscopist participated in a practice session performing retinoscopy on sheep. Measurements were performed approximately 30–45 min after cycloplegia was induced by 0.5% tropicamide (Mydramide, Fischer Pharmaceutical Labs, Israel) and 10% phenylephrine (Efrin-10, Fischer Pharmaceutical Labs, Israel) ophthalmic solutions. The sheep were alert during testing and held steady by one or two handlers. Retinoscopy was conducted using a handheld streak retinoscope, and the first eye to be refracted in each animal was determined using a computer-generated randomization table. Each eye was refracted twice. Retinoscopy was performed at a distance of 50 cm, maintained by the examiner's arm length throughout testing. Both the horizontal (180 degrees, vertical streak) and vertical (90 degrees, horizontal streak) meridians were refracted. A series of negative and positive spherical lenses (Luneau retinoscopy rack) were placed in front of the eye while the retinoscope streak was moved along the meridian, until neutrality was observed. Refractive results were recorded on a data sheet and later converted to take into account the working distance of 50 cm (2 D).

### Ocular ultrasonography

The ocular ultrasonographic examination was performed by means of B-Mode ultrasonography using a Mindray M9 portable ultrasound machine with a 5–20 MHz linear transducer (Mindray, Shenzhen, China). All examinations were performed by an Israeli Diplomate of Veterinary Radiology (IA). The investigator was masked to the genetic status of sheep in cohorts 1 and 2, but not to cohort 3. Two drops of 0.4% oxybuprocaine hydrochloride ophthalmic solution (Localin, Fischer Pharmaceutical Labs, Israel) were applied to the tested eye approximately one minute prior to the scan, ultrasonography gel was deposited on the transducer and the transducer was lightly placed on the cornea, perpendicular to the globe. A single frame was captured from each scan, imaging the cornea, anterior lens capsule, posterior lens capsule and the retina. The captured image was used to measure the distance from cornea to the anterior lens capsule (distance 1 = anterior chamber depth), cornea to the posterior lens capsule (distance 2) and cornea to retina (distance 3 = globe axial length). Lens axial length was calculated by subtracting distance 1 from distance 2 and vitreous chamber depth was calculated by subtracting distance 2 from distance 3.

### Pupil light reflex testing

The PLR was evaluated in both eyes of four Improved Awassi day-blind (cohort 1) and four Afec-Assaf WT (cohort 2) sheep. These eight animals were randomly selected from cohorts 1 & 2, respectively, excluding cohort 1 sheep that are part of an ongoing gene therapy study^36,38^. The test was performed on alert animals, manually restrained in dim light (121 lx). First, cPLR was evaluated using a commercial tester (BioMed Vision Technologies, Ames, IA, USA), which emits red (wavelength 630 nm) and blue (wavelength 480 nm) light with identical intensity of 200,000 lx. The afferent component of the PLR triggered by these wavelengths and intensities is generated by cones and ipRGCs, respectively^24,27,87^. Illumination of the right eye using red light was performed at a distance of 3 cm for 10 s. During illumination, the vertical pupil diameter of the illuminated eye was measured with an accuracy of ± 0.01 mm using a REXBETI digital caliper (REXBETI tools, Beijing, China) placed next to (but not touching) the cornea, as there is no automated pupillometry for the elliptic sheep pupil. The same procedure was repeated in the left eye with a 30 s interval between the two eyes. The two eyes were then tested using blue light with 30 s intervals between measurements. Because of the ovine pupil shape (horizontally elliptical) we did not measure horizontal pupil diameter, as it constricts minimally in response to light. Following cPLR testing, a Finoff transilluminator (Welch Allyn Medical Products, Skaneateles Falls, NY, USA) was used to measure standard PLR response to white light (12,845 lx) in both eyes of each sheep. The investigator measuring pupil size was not masked, and order of sheep was not randomized.

### Statistical analysis

Statistical analysis was conducted using JMP Pro 14.0.0 (SAS institute Inc., 2016. Cary, NC, USA). The Shapiro–Wilk test, used to assess the normality of the distribution of data, determined that the data are not normally distributed. Therefore, the Kruskal–Wallis 1-way ANOVA nonparametric test was used to compare refractive errors, ultrasonographic measurements and vertical pupil diameters between cohorts of sheep.

The mean of the two refractive error measurements in each meridian of each eye was calculated. The spherical equivalent (SE) refractive error was calculated in "minus cylinder" form by subtracting half of the difference between the measurements of the two meridians, to the more negative meridian. Spearman’s correlation was used to analyze the relationship between refractive errors, ultrasonographic measurements and vertical pupil diameter in left and right eyes. The horizontal and vertical meridians' and SE refractive errors and the ultrasound findings of the left eyes were compared using the Kruskal–Wallis 1-way ANOVA nonparametric test. Repeated measures ANOVA was used to compare vertical pupil diameters in response to blue, red and white light within cohorts 1 & 2.

Values were considered significant for p < 0.05. In cases of significance, the Mann–Whitney nonparametric test was used for pairwise comparisons, and significance for these tests was set according to the Bonferoni correction at P < 0.017.

## Data Availability

The datasets generated during and/or analyzed during the current study are available from the corresponding author on reasonable request.
